# Supplementary material for: WordCommentsAnalyzer: A windows software tool for qualitative research
Source: F1000Res. 2018 Sep 4;7:536. Originally published 2018 May 3. [Version 2] doi: 10.12688/f1000research.14819.2 (PMC6137411; doi:10.12688/f1000research.14819.2)
Supplement: Supplementary file 3 [file f1000research-7-17614-s0002.tgz › b2a79496-4ada-47c4-9682-a99ce867836d.zip!/Eight Iranian Health Journals/Journal of Research in Rehabilitation Sciences _7.docx]

© Journal of Research in Rehabilitation Sciences . This work is licensed under CC BY-NC 4.0 (https://creativecommons.org/licenses/by-nc/4.0/).

Volume & Issue: دوره 12، شماره 4: 1395

Title: مقایسه میزان سرعت و درک خواندن دانش‌آموزان کاشت حلزون شده و شنوای پایه اول دبستان

http://jrrs.mui.ac.ir/index.php/jrrs/article/view/2684

مقدمه: مهم‌ترین حس در رشد طبیعی گفتار و زبان، شنوایی است و ناشنوایان بیشترین آسیب ارتباطی را نشان می‌دهند. از آن‌جا که رشد طبیعی گفتار و زبان در دوران کودکی، پیش‌نیازهای لازم جهت رشد خواندن را فراهم می‌کند، آسیب‌های خواندن در افراد ناشنوا به فراوانی مشاهده می‌گردد. انجام کاشت حلزون شنوایی با فراهم آوردن درون‌داد شنوایی و بهبود رشد گفتار و زبان، می‌تواند باعث دستیابی به پیش‌نیازهای خواندن و کاهش مشکلات خواندن شود. پژوهش حاضر با هدف بررسی وضعیت خواندن کودکان کاشت حلزون شده انجام شد. مواد و روش‌ها: در این مطالعه، خواندن متن در 11 کودک کاشت حلزون شده پایه اول دبستان مورد بررسی قرار گرفت و میانگین سرعت خواندن و درصد پاسخ‌های درک خواندن آنان با 11 دانش‌آموز طبیعی با استفاده از آزمون‌های آماری مقایسه گردید. یافته‌ها: نتیجه آزمون Independent t در مورد هر دو متغیر سرعت و درصد پاسخ‌های درک خواندن، نشان داد که تفاوت معنی‌داری بین میانگین‌ها در دانش‌آموزان کاشت حلزون شده و طبیعی وجود داشت (001/0 > P). نتیجه‎گیری: نتایج پژوهش نشان می‌دهد که کودکان کاشت حلزون شده نسبت به همتایان شنوای خود از سال ابتدایی تحصیل با مشکلاتی در خواندن سریع و روان و درک مطالب نوشته شده مواجه هستند که به نظر می‌رسد ناشی از نقایص زبانی آن‌ها باشد. با این وجود، تفاوت‌های فردی زیادی در بین این کودکان مشاهده می‌شود.

Title: اثربخشی درمان متمرکز بر کاربردشناسی زبان

http://jrrs.mui.ac.ir/index.php/jrrs/article/view/2720

مقدمه: مهارت‌های مکالمه‌ای، مهم‌ترین مهارت‌های کاربردشناسی زبان هستند. در میان مهارت‌های مکالمه‌ای، اصلاح مکالمه و درخواست توضیح در جلوگیری از شکست‌های ارتباطی نقش مهمی ایفا می‌کنند. از جمله اختلالاتی که نقایص برجسته کاربردشناسی زبان دارد، کودکان دارای اختلال ارتباط اجتماعی (Social communication disorder یا SCD) هستند که به دلیل نقص در مهارت‌های کاربرد شناختی و مکالمه‌ای، به طور مکرر با شکست‌های ارتباطی مواجه می‌شوند. بنابراین، ارایه درمان‌هایی که بر مهارت‌های مکالمه‌ای این کودکان متمرکز باشد، از اهمیت خاصی برخوردار است. هدف از انجام مطالعه حاضر، بررسی تأثیر درمان متمرکز بر مهارت‌های اصلاح مکالمه و درخواست توضیح در کودکان دارای SCD بود. مواد و روش‌ها: این مطالعه با طرحی مورد منفرد و به شیوه Multiple probe، بر روی سه کودک 5 تا 7 ساله دارای SCD مراجعه‌ کننده به کلینیک گفتار درمانی انجام شد. آزمودنی‌ها از سه جلسه قبل تا سه جلسه بعد از درمان مورد ارزیابی قرار گرفتند و انواع استراتژی‌های اصلاح مکالمه و درخواست توضیح طی 12 جلسه درمانی 40 دقیقه‌ای به آنان آموزش داده شد. داده‌ها بر اساس تحلیل چشمی نمودارهای رسم شده برای هر کودک در مهارت‌های ذکر شده و با استفاده از مقیاس IRD (Improvement rate difference) مورد تجزیه ‌و تحلیل قرار گرفت. یافته‌ها: تحلیل چشمی نمودارها و IRD نشان داد که همه آزمودنی‌ها در مهارت‌های اصلاح مکالمه و درخواست توضیح پیشرفت داشتند (برای مهارت اصلاح مکالمه IRD بیشتر از 91 درصد و برای درخواست توضیح  ID بیشتر از 83 درصد) و می‌توانستند انواع استراتژی‌های اصلاح مکالمه و درخواست توضیح را به کار ببرند. نتیجه‎گیری: اثربخشی برنامه درمانی متمرکز بر مهارت‌های اصلاح مکالمه و درخواست توضیح در کودکان دارای SCD قابل‌ قبول بود.

Title: اثربخشی روش‌های یکپارچه‌‌سازی حسی- حرکتی بر خام حرکتی کودکان مبتلا به ناتوانی یادگیری غیرکلامی

http://jrrs.mui.ac.ir/index.php/jrrs/article/view/2695

مقدمه: یکی از مهم‌ترین مشکلات کودکان مبتلا به ناتوانی یادگیری غیر کلامی، خام حرکتی و هماهنگی ضعیف حسی- حرکتی می‌باشد که مانع دستیابی کودکان به پتانسیل‌های تحصیلی و پیشرفت هیجانی و اجتماعی می‌شـود. پژوهش حاضر، با هدف بررسی اثربخشی روش‌های یکپارچه‌سازی حسی- حرکتی بر خام حرکتی کودکان مبتلا به ناتوانی یادگیری غیر کلامی انجام گرفت. مواد و روش‌ها: روش مطالعه طرح آزمایشی پیش‌آزمون- پس‌آزمون بود. بدین منظور، 7 کودک دارای ناتوانی یادگیری غیر کلامی (4 پسر و 3 دختر) در پایه‌های تحصیلی دوم تا پنجم دبستان شهرستان‌های تهران با استفاده از روش نمونه‌گیری غیر تصادفی از نوع قضاوتی انتخاب شدند. مقیاس حرکتی Lincoln-Oseretsky قبل و بعد از انجام مداخله اجرا گردید و داده‌های پژوهش از طریق آزمون Wilcoxon مورد تجزیه و تحلیل قرار گرفت. یافته‌ها: برنامه یکپارچه‌سازی حسی- حرکتی بر مهارت‌های حسی- تعادلی (017/0 = P)، حرکتی درشت (018/0 = P)، حرکتی ظریف (016/0 = P)، هماهنگی دو طرفه (012/0 = P) و نمره کل خام حرکتی (016/0 = P) تأثیر مثبت و معنی‌داری داشت. نتیجه‎گیری: یکپارچه‌‌سازی حسی- حرکتی تفاوت معنی‌داری در خام حرکتی کودکان مبتلا به ناتوانی یادگیری غیر کلامی ایجاد می‌کند و موجب بهبود مهارت‌های حرکتی آن‌ها در چهار حیطه تعادل، مهارت‌های درشت، ظریف و هماهنگی حرکتی دو طرفه می‌شود.

Title: تأثیر آموزش مهارت‌های اجتماعی بر نشانه‌های اختلال طیف اوتیسم در نوجوانان: یک مطالعه نیمه تجربی

http://jrrs.mui.ac.ir/index.php/jrrs/article/view/2751

مقدمه: از مهم‌ترین مشکلات افراد مبتلا به اختلال طیف اوتیسم، نقص در مهارت‌های اجتماعی است که عملکرد روزانه فرد را مختل یا محدود می‌کند. هدف از انجام پژوهش حاضر، بررسی اثربخشی آموزش مهارت‌های اجتماعی بر نشانه‌های اوتیسم در نوجوانان بود. مواد و روش‌ها: این پژوهش به روش نیمه تجربی همراه با پیش‌آزمون- پس‌آزمون انجام شد. جامعه آماری مطالعه شامل 60 نفر از نوجوانان مبتلا به اختلال طیف اوتیسم با عملکرد بالا در شهر تهران بود که با روش نمونه‌گیری هدفمند، 12 نفر انتخاب شدند و در 10 جلسه 90 دقیقه‌ای (هفته‌ای دو جلسه) شرکت نمودند و پس از آن با استفاده از مقیاس Gilliam Autism Rating Scale (GARS) مورد ارزیابی قرار گرفتند. داده‌ها با استفاده از آزمون تحلیل واریانس اندازه‌گیری‌های‌ مکرر تجزیه و تحلیل گردید. یافته‌ها: آموزش مهارت‌های اجتماعی تأثیر معنی‌داری بر کاهش رفتارهای کلیشه‌ای (110/0 = P)، مشکل در برقراری ارتباط (260/0 = P)، تعاملات اجتماعی (080/0 = P) و علایم اختلالات رشدی (770/0 = P) نداشت. نتیجه‎گیری: آموزش مهارت‌های اجتماعی به ‌تنهایی تأثیر چندانی در علایم اختلال طیف اوتیسم نوجوانان ندارد. بنابراین، آموزش‌هایی که سایر حیطه‌های دارای مشکل‌ این نوجوانان را پوشش می‌دهد، برای کاهش علایم این اختلال توصیه می‌شود.

Title: پیش‌بینی نمای قوس ساجیتال کفش‌های غلتکی بر اساس کینماتیک مچ پا حین راه رفتن: رویکرد شبکه عصبی مصنوعی

http://jrrs.mui.ac.ir/index.php/jrrs/article/view/2734

مقدمه: زیره‌های غلتکی به صورت نمای قوس‌دار در صفحه ساجیتال، یکی از رایج‌ترین اصلاحات درمانی در کفش‌های غلتکی به منظور تغییر یا تطابق با کینماتیک و کینتیک مفاصل اندام تحتانی می‌باشد. هرچند معیار تجویز این قوس‌ها بر اساس ملاحظات تئوری صورت می‌گیرد، انجام مطالعات تجربی و آزمون و خطا می‌تواند به تجویز مناسب‌تر و استفاده بهتر از آن‌ها کمک کند. رویکرد مکمل، استفاده از تکنولوژی‌های هوشمند به منظور پیش‌بینی نمای قوس جهت تطابق یافتن با وضعیت هر مفصل است. هدف از انجام مطالعه حاضر، پیش‌بینی نمای قوس کفش‌های غلتکی در صفحه ساجیتال بر اساس کینماتیک مچ پا طی راه رفتن با استفاده از تکنولوژی شبکه عصبی مصنوعی بود. مواد و روش‌ها: در این مطالعه، 20 فرد سالم (با میانگین سنی 1/33 سال) در دو وضعیت استفاده از دو نوع کفش مختلف با دو نوع نمای قوس متفاوت در یک مسیر مستقیم 10 متری راه رفتند و کینماتیک مچ پای آنان با استفاده از نشانگرهای انعکاسی ثبت گردید. به شبکه عصبی مصنوعی آموزش داده شد تا حرکات مچ پا در صفحه ساجیتال در فاز استانس راه رفتن را با نمای قوس زیره‌ غلتکی مورد استفاده تطبیق دهد و سپس بتواند نوع قوس بعدی را بر اساس اطلاعات قبلی داده شده به آن پیش‌بینی کند. به منظور آموزش شبکه عصبی، داده‌های حاصل از آنالیز حرکات مچ پای 13 نفر از شرکت کنندگان و نمای قوس زیره‌ غلتکی کفش آن‌ها استفاده گردید (گروه شاهد) و از داده‌های دیگر شرکت کنندگان به منظور اعتباربخشی به اهداف مطالعه استفاده شد (گروه مورد). یافته‌ها: دقت به دست آمده از آنالیز داده‌ها بسیار رضایت‌بخش بود؛ چرا که ضریب همبستگی بین یافته‌های پیش‌بینی شده و نمای واقعی قوس در داده‌های حاصل از گروه مورد برای هر دو نوع کفش غلتکی مورد استفاده در مطالعه، بیشتر از 95/0 حاصل شد. نتیجه‎گیری: در مطالعه حاضر یک الگوریتم جدید جهت مشخص کردن ویژگی‌های کفش غلتکی با استفاده از مدل شبکه عصبی به دست آمد. نتایج حاصل شده برای طراحان کفش، ارتوزها و پروتزهای اندام تحتانی و گچ/ چکمه‌های پیاده‌روی قابلیت استفاده دارد.

Title: ارزیابی پوسچر سر و تنه مردان مبتلا به سردرد با منشأ گردن از طریق عکاسی: یک مطالعه‌ مقدماتی

http://jrrs.mui.ac.ir/index.php/jrrs/article/view/2753

مقدمه: سردرد با منشأ گردن (Cervicogenic headache)، یکی از انواع سردردهای مزمن و مکرر است که در اثر اختلالات عضلانی- اسکلتی گردن به وجود می‌آید. با توجه به مشکلات عضلانی مشاهده شده در بیماران مبتلا به سردرد با منشأ گردن، ارتباط اختلال عملکرد عضلات گردنی با پوسچر سر و گردن و ارتباط بین انحناهای ستون ‌فقرات و راستای لگن، ارزیابی پوسچر در این بیماران اهمیت می‌یابد. بنابراین، هدف از انجام مطالعه حاضر، ارزیابی پوسچر استاتیک ستون ‌فقرات و لگن با بررسی زوایای مختلف از طریق عکاسی در حالت ایستاده بود. مواد و روش‌ها: این مطالعه مورد- شاهدی بر روی 10 مرد مبتلا به سردرد با منشأ گردن و 10 مرد سالم با سن و شاخص توده بدنی (Body mass index یا BMI) همسان انجام شد. با استفاده از یک دوربین دیجیتال، از نماهای خلفی، قدامی و کناری آزمودنی‌ها عکس‌برداری با سه بار تکرار صورت گرفت و سپس زوایای پوسچرال با استفاده از نرم‌افزار AutoCAD اندازه‌گیری گردید. یافته‌ها: از بین زوایای اندازه‌گیری شده، میانگین زاویه‌ گردن (2T-2C) (022/0 = P)، راستای افقی سر (001/0 = P)، راستای افقی اسکاپولاها (001/0 > P)، راستای افقی خارهای خاصره قدامی فوقانی (031/0 = P) و راستای افقی خارهای خاصره خلفی فوقانی (038/0 = P) بین دو گروه تفاوت معنی‌داری داشت. پایایی درون آزمونگر در همه‌ زوایای پوسچرال اندازه‌گیری گردید و مقدار بالا و قابل ‌قبولی [72/0 < Intraclass Correlation Coefficient  (ICC)] به دست آمد. نتیجه‎گیری: به نظر می‌رسد پوزیشن ناحیه‌ فوقانی ستون‌ فقرات گردنی مردان مبتلا به سردرد با منشأ گردن در مقایسه با افراد سالم تفاوت دارد و مهره‌ دوم گردنی نسبت به ستون فقرات گردنی- سینه‌ای جلوتر قرار گرفته است. از این‌رو، زاویه‌ گردن می‌تواند شاخص خوبی برای نشان دادن راستای قرارگیری مهره‌ دوم گردنی از طریق عکاسی با دوربین دیجیتال باشد.

Title: مقایسه تأثیر دو روش درمانی سوزن خشک و آموزش اصلاح پاسچر بر پارامترهای درد، ناتوانی عملکردی گردن و آستانه فشاری در بیماران مبتلا به سندرم نقاط ماشه‌ای فعال عضله تراپزیوس فوقانی: مطالعه کارآزمایی بالینی تصادفی (مطالعه مقدماتی)

http://jrrs.mui.ac.ir/index.php/jrrs/article/view/2760

مقدمه: نقطه ماشه‌ای در عضله تراپزیوس فوقانی، یافته شایعی در افراد مبتلا به دردهای ناحیه گردن است. مطالعه حاضر با هدف مقایسه تأثیر دو روش درمانی سوزن خشک (Dry needling) و اصلاح پاسچر بر پارامترهای درد، ناتوانی عملکردی گردن (Neck Disability Index یا NDI) و آستانه فشاری (Pressure threshold یا PT) در شرکت کنندگان مبتلا به سندرم نقاط ماشه‌ای عضله تراپزیوس فوقانی صورت گرفت. مواد و روش‌ها: این مطالعه از نوع کارآزمایی بالینی و یک سوکور بود که در آن 15 شرکت کننده به صورت تصادفی در سه گروه قرار گرفتند. گروه اول درمان سوزن خشک و فیزیوتراپی رایج، گروه دوم آموزش اصلاح پاسچر و فیزیوتراپی رایج و گروه سوم (گروه شاهد) فقط فیزیوتراپی رایج دریافت نمودند. اثرات درمانی از سه جنبه PT (آلگومتر)، شدت درد (Visual analogue scale یا VAS) و NDI طی سه مرحله (پایه، جلسه آخر و 6 هفته بعد از درمان) مقایسه گردید. یافته‌ها: با وجود اثرات معنی‌دار هر یک از روش‌های درمانی در افزایش PT، کاهش شدت درد و NDI (050/0 > P)، اما هیچ کدام از روش‌های درمانی در جلسه آخر نسبت به دیگری برتری معنی‌داری نداشت (050/0 < P)؛ در حالی که 6 هفته پس از درمان، دو گروه سوزن خشک و اصلاح پاسچر تغییرات معنی‌داری را نسبت به گروه شاهد نشان دادند (050/0 > P). توان آزمون آماری در متغیر VAS در آخرین جلسه و 6 هفته بعد از درمان به ترتیب 68 و 79 درصد بود. نتیجه‎گیری: با توجه به تأثیرات مثبت گروه‌های درمانی در طی زمان و تفاوت معنی‌دار دو گروه درمانی سوزن خشک و اصلاح پاسچر نسبت به گروه شاهد 6 هفته بعد از درمان، استفاده از این دو روش درمانی در شرکت کنندگان مبتلا به نقاط ماشه‌ای فعال عضله تراپزیوس فوقانی توصیه می‌شود.

Title: مرور نظامند بر تأثیر سن و محرک‌های گفتاری بر نمرات نیزالانس افراد طبیعی

http://jrrs.mui.ac.ir/index.php/jrrs/article/view/2640

مقدمه: ارزیابی‌های دستگاهی به دلیل داشتن پتانسیل برای ارزیابی کمی تشدید خیشومی، مورد توجه می‌باشد. یکی از این ابزارها نیزومتر (Kay elemetrics, Lincoln park, NJ) است. نیزومتر با اندازه‌گیری انرژی آکوستیک دهان و بینی در طی تولید گفتار و محاسبه نمره نیزالانس یک ارزیابی دستگاهی از نیزالیتی ارایه می‌دهد. مطالعه حاضر با مروری هدفمند بر آخرین مقالات و کتاب‌های منتشر شده در زمینه نیزالانس، به بررسی تأثیرات سن و محرک‌های گفتاری بر نمرات نیزالانس پرداخت. مواد و روش‌ها: با استفاده از کلمات کلیدی "نیزالانس"، "نیزومتر"،" نمره هنجار نیزالانس"، "ارزیابی دستگاهی اختلالات تشدید" مقالات مرتبط در بازه زمانی سال‌های 1990 تا 2016 از پایگاه‌های اطلاعاتی ASHA، Pub Med، Science direct، Google scholar، Pro Quest، Cochrane، Wiley، SID، Iran Medex و Iran Doc استخراج شد. یافته‌ها: تعداد 67 مقاله از پایگاه‌های ذکر شده به دست آمد و با توجه به معیارهای ورود و خروج، 38 مقاله که به بررسی تأثیر سن و جنس بر نمرات نیزالانس پرداخته بود، مورد بررسی قرار گرفت. با توجه به تحقیق انجام شده در رابطه با تأثیر سن و محرک گفتاری در مطالعات مختلف نتایج متفاوتی به دست آمد. نتیجه‎گیری: با توجه به این که علاوه بر سن و محرک گفتاری عوامل دیگری همچون زبان، لهجه، جنس و ... هم از عوامل تأثیرگذار بر نیزالانس است، انجام یک مطالعه فرا تحلیلی جهت مشخص کردن تأثیر هر کدام از عوامل لازم می‌باشد.
